# Spread of oxazolidinone-resistant enterococci in an Italian pig slaughterhouse

**DOI:** 10.3389/fmicb.2026.1894422

**Published:** 2026-07-23

**Authors:** Francesca Romana Massacci, Elisa Albini, Lucilla Cucco, Elisa Russo, Maria Elena Nigro, Giorgia Binucci, Sonia Nina Coccitto, Marzia Cinthi, Serena Simoni, Eleonora Scoccia, Carmen Maresca, Eleonora Giovanetti, Giovanni Pezzotti, Chiara Francesca Magistrali, Andrea Brenciani

**Affiliations:** 1 Istituto Zooprofilattico Sperimentale dell’Umbria e Delle Marche ‘Togo Rosati’, Perugia, Italy; 2 Department of Biomedical Sciences and Public Health, Unit of Microbiology, Polytechnic University of Marche Medical School, Ancona, Italy; 3 Department of Life and Environmental Sciences, Unit of Microbiology, Polytechnic University of Marche, Ancona, Italy; 4 Istituto Zooprofilattico Sperimentale della Lombardia e dell'Emilia Romagna "Bruno Ubertini", Brescia, Italy

**Keywords:** antibiotic-resistance, *Enterococcus faecalis*, *Enterococcus faecium*, mobile genetic elements, oxazolidinone

## Abstract

The swine production chain represents a critical point for the spread of antimicrobial resistance, with slaughterhouses acting as potential hotspots for cross-contamination. The veterinary use of phenicols may promote resistance to oxazolidinones, a last-resort class of antibiotics. This study investigated the presence of oxazolidinone-resistant enterococci in a pig slaughterhouse in central Italy, analyzing 376 samples, 25.8% of which tested positive for *Enterococcus* spp. resistant to florfenicol. Environmental samples collected at the end of the day of slaughter showed higher contamination levels compared to those collected beforehand. PCR analysis of 42 isolates of *Enterococcus faecalis* and *Enterococcus faecium* revealed a high prevalence of the *optrA* gene, followed by *poxtA* and *cfr*(D). The data indicated high resistance rates to tetracycline and erythromycin, consistent with existing literature. Linezolid resistance was observed in 73.8% of isolates, while no vancomycin resistance was detected. Whole Genome Sequencing (WGS) analysis revealed diverse sequence types, complex resistomes, multiple virulence factors, and considerable variability in the genetic elements carrying *cfr*/*cfr*(D), *optrA*, and *poxtA*. These determinants were often plasmid-borne and co-localized with other antibiotic and heavy metal resistance genes, which could contribute to their persistence and dissemination through co-selection mechanisms. Overall, the findings highlight the widespread occurrence of oxazolidinone-resistant enterococci in the pig production chain and their potential circulation across different production stages. An integrated approach encompassing prudent antibiotic use in livestock, good hygiene practices at the slaughterhouses, and increased awareness of the problem will be essential to mitigate the spread of antimicrobial resistance along the food production chain.

## Introduction

Antimicrobial resistance (AMR) represents one of the major global public health challenges (EFSA, 2024). This phenomenon, which affects humans, animals, and the environment, requires a multidisciplinary approach grounded in the “One Health” concept to ensure effective management. Different studies have demonstrated that antibiotic-resistant bacteria (ARB) and antibiotic resistance genes (ARGs) can be transferred from livestock to humans, posing a potential public health risk (Wee et al., 2020; EFSA, 2024). The swine production chain is considered a critical point for the emergence and dissemination of AMR, from primary production to the final product intended for human consumption. Pigs may act as a reservoir of ARB, and both animal transport and lairage facilities have been identified as risk factors for their spread (Koutsoumanis et al., 2022). Furthermore, slaughterhouses represent environments where cross-contamination between carcasses and the dissemination of ARB and ARGs can occur, thereby increasing the risk of resistance transmission to humans and the environment (Savin et al., 2020b, 2020a). However, information regarding the presence of ARB in swine carcasses and slaughterhouse environments remains limited.

Enterococci are ubiquitous Gram-positive bacteria commonly present in the intestinal microbiota of humans and animals. Although part of the commensal flora, some species, particularly *Enterococcus faecium* and *Enterococcus faecalis*, are recognized as opportunistic pathogens responsible for severe infections in humans, including endocarditis, bacteremia, urinary tract infections, and intra-abdominal infections (Arias and Murray, 2012). The ability of enterococci to acquire and transmit antibiotic resistance represents a significant challenge to antimicrobial therapy, making infection management increasingly complex. Oxazolidinones, such as linezolid, are considered last-resort antibiotics for the treatment of multidrug-resistant enterococcal infections (Brenciani et al., 2022).

Although oxazolidinones are not authorized for veterinary use, increasing evidence indicates a rise in linezolid resistance among enterococci isolated from food-producing animals (Fioriti et al., 2020; Schwarz et al., 2021; Brenciani et al., 2022; Coccitto et al., 2022). Recent European studies have also highlighted the occurrence of linezolid-resistant enterococci in livestock and food-producing animals, underlining the need for continuous monitoring of these resistance determinants within a One Health framework. The occurrence of oxazolidinone-resistant enterococci in livestock raises concerns about potential transmission along the food chain, with significant implications for public health. Several studies suggest that the use of phenicols in veterinary medicine may exert selective pressure favoring the spread of oxazolidinone resistance in enterococci (Wang et al., 2020; Brenciani et al., 2022). In particular, specific genetic determinants, such as the *cfr*, *optrA*, and *poxtA* genes, confer resistance to both phenicols and oxazolidinones, posing a risk of co-selection of resistant strains in environments characterized by intensive use of these antimicrobials (Brenciani et al., 2022; Schwarz et al., 2021). For this reason, florfenicol-resistant enterococci were selected as the primary screening target in the present study, as resistance to florfenicol may be associated with the presence of transferable oxazolidinone resistance determinants.

In this scenario, the surveillance of oxazolidinone resistance in animal-derived enterococci is essential to assess the potential risk to human health.

The present study investigated the occurrence of oxazolidinone-resistant enterococci in a swine slaughterhouse located in central Italy. *E. faecium* and *E. faecalis* isolates were characterized by the presence of oxazolidinone resistance genes, with the aim of evaluating the risk associated with the occurrence of these bacteria in a pig slaughterhouse.

## Materials and methods

### Sampling

This study was carried out from April 2023 to March 2024 in a slaughterhouse located in Central Italy, with a capacity of 2,500 pigs per day, operating for 4 days per week, with all animals sourced from national farms. The slaughterhouse was equipped with lairage facilities.

The study design considered an assumed prevalence of antibiotic-resistant bacteria of 50%, a confidence level of 95%, and a precision of 5%. Based on these assumptions, a longitudinal sampling scheme was implemented to monitor the occurrence of florfenicol-resistant enterococci throughout a one-year period. Sampling was performed with a quarterly frequency, once per season (spring: April 2023; summer: August 2023; autumn: October 2023; winter: January 2024), to ensure temporal coverage of potential seasonal variations. On each slaughter day, 33 cecal content samples and 33 carcass swabs were collected.

On each slaughter day, 22 environmental samples were collected at the beginning of each sampling day before the first pig was slaughtered. Sampling was carried out using pre-moistened sponges with buffered peptone water (Spongebags, Solar Biologicals Inc.), for eleven different points along the slaughter line both before and after slaughtering (scalding tanks, carcass chute, hooks, knives, evisceration sink, control panel, inspection table, operators’ aprons, organ trays, drain outlets, and cold room door gaskets). Additionally, four post-slaughter environmental samples were collected from the lairage area (doors, floors, windowsills, and pen dividers) using pre-moistened sponges with buffered peptone water swabbed on surfaces.

On each slaughter day, pigs were selected by simple randomization. Cecal contents and carcass swabs were taken from each pig. Carcass swabs were taken before the final washing, according to the UNI EN ISO 17604:2003/E (2003) procedure. Briefly, five different points on each half carcass (distal hind limb, hind limb, lateral abdomen, medial abdomen, mid-dorsal region) were sampled using 100 cm^2^ sterile square templates and pre-moistened sponges. Five hundred cm^2^ were sampled from each half carcass, corresponding to a total sampled area of 1,000 cm^2^ per carcass.

All samples were immediately placed in sterile containers, maintained at 4 °C until processing, and cultured at the arrival in laboratory.

### Microbiological culture

The microbiological analysis of cecal contents, carcass swabs and environmental samples was carried out to detect florfenicol-resistant enterococci. All samples were subjected to a pre-enrichment step in buffered peptone water (APT) at a 1:10 ratio, homogenized, and incubated at 37 °C for 24 h. Subsequently, 10 μL of the culture was plated onto Slanetz–Bartley agar supplemented with florfenicol (10 mg/L) and incubated at 37 °C for 48 h. Colonies of interest were sub-cultured on blood agar (5% sheep red blood cells), incubated at 37 °C, and identified using MALDI-TOF mass spectrometry (MALDI Biotyper, Bruker Daltonics).

### Antimicrobial susceptibility testing

To study the antibiotic resistance of *E. faecalis* and *E. faecium* isolates, a representative subset of isolates was selected. Isolates were selected according to predefined epidemiological criteria to maximize the representation of different farms and sample matrices while avoiding the overrepresentation of closely related strains. Specifically, only one *E. faecalis* or *E. faecium* isolate per matrix (cecal content or carcass) from the same farm of origin was included. This selection resulted in 42 *E. faecalis* and *E. faecium* isolates, including 34 *E. faecalis* and 8 *E. faecium* isolates.

Antimicrobial susceptibility was determined using the broth microdilution method with a commercial 96-well MIC panel (CMV3AGPF Sensititre; Trek Diagnostic Systems Inc., England), following the manufacturer’s instructions. Additional susceptibility testing for tedizolid was performed using E-test strips (Liofilchem, Roseto degli Abruzzi, Italy). *E. faecalis* ATCC 29212 was used as a control strain. MIC values were interpreted according to EUCAST clinical breakpoints (EUCAST version 16.0, 2026) and CLSI breakpoints for *Enterococcus* spp. (CLSI, 2024b, 2024a).

### Molecular analysis

Genomic DNA was extracted using the QIAamp DNA Mini Kit (Qiagen Inc., Hilden, Germany). Enterococcal isolates were screened by PCR for the presence of both the oxazolidinone-resistance genes (*cfr* and *cfr*-like, *optrA*, and *poxtA*) (Cinthi et al., 2022b) using primer pairs previously described (Fioriti et al., 2020). The *poxtA*-carrying *E. faecium* EF3 (Fioriti et al., 2021), the *cfr*-, *optrA*-carrying *E. faecium* E35048 (Morroni et al., 2018) were used as positive controls in PCR experiments.

### Whole genome sequencing and bioinformatic analysis

In order to characterize the sequence type and the genetic element responsible for the spread of antibiotic resistance genes, extracted DNA of the 42 enterococci was subjected to WGS with two approaches: Illumina MiSeq platform (Illumina®, San Diego, CA, USA) using a 2 × 150 bp paired-end technology and a long-read sequencing approach (MinION, Oxford Nanopore Technologies, Oxford, UK). Hybrid assembly was performed with Unicycler v.0.4.8.^1^ After trimming and quality assessment, the reads were assembled with SPAdes genome assembler v3.11.1 (Prjibelski et al., 2020) and annotated using Prokka v1.14.6 (Seemann, 2014). Core genome alignments were generated with Roary v3.11.3 (Page et al., 2015) and used to construct maximum-likelihood phylogenetic trees with FastTree 2.1.11 (Price et al., 2010).

Sequence types (STs) were assigned using the *E. faecalis* and *E. faecium* MLST databases.^2^

*In silico* identification of acquired antimicrobial resistance genes and ribosomal mutations involved were carried out using dedicated tools available at the Center for Genomic Epidemiology available at http://www.genomicepidemiology.org (e.g., MLST v.2.0, ResFinder 4.1, LRE-finder 1.0, Virulencefinder 2.0, PlasmidFinder 2.1, CSI Phylogeny 1.4) and by the BLAST suite^3^ and considering only genes with a ≥ 90% coverage and ≥90% identity. The BLAST Ring Image Generator (BRIG) sotware was used to compare relevant genetic elements. The RAST tool^4^ was used for the annotation of DNA sequences.

## Results and discussion

### Isolation, distribution of isolates and detection of oxazolidinone resistance genes in florfenicol-resistant enterococci

A total of 376 samples were collected, of which 97 (25.8%) tested positive for florfenicol-resistant *Enterococcus* spp. (Table 1). Florfenicol-resistant Enterococcus spp. were detected during all four sampling campaigns (spring, summer, autumn, and winter), indicating a persistent occurrence throughout the study period. No apparent seasonal trend was observed in the distribution of resistant isolates or resistance determinants. For environmental samples, a significant difference was observed between those collected before the start of slaughtering operations and those collected at the end (Chi-square test: *p* < 0.05). The matrices most frequently contaminated were those located at the beginning of the slaughter line. Scalding tanks, carcass slides, and drain outlets tested positive prior to slaughtering activities. Environmental samples collected before the start of slaughtering activities were obtained after the routine cleaning and disinfection procedures performed at the end of the previous working day, whereas post-operational samples were collected at the end of slaughtering activities before the subsequent sanitation procedures. The significantly higher contamination observed in post-operational samples indicates that slaughtering activities contributed to the dissemination of florfenicol-resistant enterococci within the processing environment. Potential contamination pathways include the release of intestinal contents during processing and the subsequent transfer of bacteria to carcasses, equipment, drains, and contact surfaces. The limited number of positive samples detected before the start of daily operations suggests that routine cleaning and disinfection procedures were generally effective in reducing environmental contamination, although some environmental niches, particularly scalding tanks, carcass slides, and drain outlets, remained contaminated and may represent potential reservoirs of resistant enterococci. A total of 114 *Enterococcus* isolates were identified to species level, as some samples were contaminated by more than one species (Table 2). Notably, *E. faecalis* predominated in carcass and environmental samples, while such predominance was not observed in cecal samples. This difference may reflect either higher initial microbial loads or greater environmental persistence of this species, as previously described (Sadowy and Luczkiewicz, 2014). The predominance of *E. faecalis* outside the intestinal compartment suggests that this species may be better adapted to survive and persist under slaughterhouse environmental conditions than other enterococci. Its frequent recovery from both carcasses and environmental surfaces may also reflect cross-contamination events occurring during processing. Similar findings have been reported in food-production environments, where *E. faecalis* is often the predominant enterococcal species recovered from equipment, processing surfaces, and animal-derived foods, likely owing to its remarkable tolerance to environmental stresses and disinfectants (Sadowy and Luczkiewicz, 2014).

**Table 1:** Florfenicol-resistant *Enterococcus* spp. positive samples, categorized by matrix of origin.

Matrix		Positive samples/total (%)
Cecum		26/132 (19.7)
Carcass		43/132 (32.6)
Environmental	Before slaughter	4/50 (8)
	After slaughter	12/46 (26.1)
	Lairage area	12/16 (75)

**Table 2:** *Enterococcus* species resistant to florfenicol based on the matrix of origin.

Matrix	Number of isolates	Bacterial species	Number/total isolates (%)
Cecum	28	*E. faecalis*	6/28 (21.4)
		*E. hirae*	6/28 (21.4)
		*E. avium*	6/28 (21.4)
		*E. faecium*	5/28 (17.8)
		*E. durans*	2/28 (7.1)
		*E. asini*	2/28 (7.1)
		*E. thailandicus*	1/28 (3.6)
Carcass	49	*E. faecalis*	33/49 (67.3)
		*E. avium*	9/49 (18.4)
		*E. hirae*	4/49 (8.1)
		*E. faecium*	2/49 (4.0)
		*E. gallinarum*	1/49 (2.0)
Environmental	37	*E. faecalis*	20/37 (54.0)
		*E. faecium*	9/37 (24.3)
		*E. avium*	3/37 (8.1)
		*E. hirae*	3/37 (8.1)
		*E. durans*	1/37 (2.7)
		*E. gallinarum*	1/37 (2.7)

### Antimicrobial susceptibility profiles and linezolid resistance genes content

**Figure 1:**
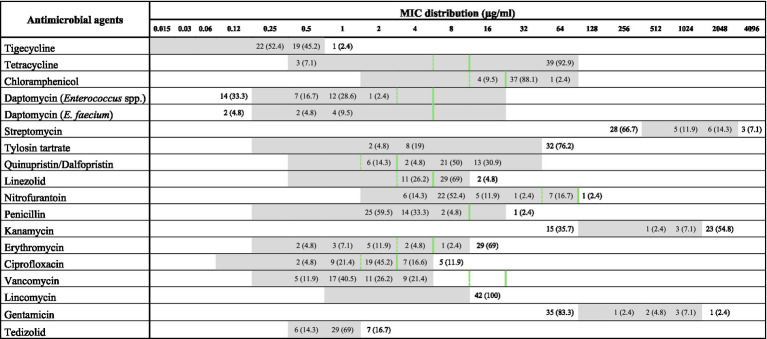
Distribution of MIC values among the 42 enterococcal isolates. Percentages are shown in brackets. Shades areas show the range of values tested for each antibiotic. Vertical green bars indicate the threshold values for clinical resistance, while dotted lines indicate intermediate susceptibility according to CLSI.

Figure 1 reports the distribution of MIC values for the 42 isolates against antimicrobial agents. All isolates were susceptible to vancomycin and daptomycin. High resistance rates were observed for tetracycline (*n* = 439, 92.9%), chloramphenicol (*n* = 38, 90.5%), linezolid (*n* = 31, 73.8%), and erythromycin (*n* = 30, 71.4%). Nearly one-third of isolates (*n* = 12, 28.5%) were resistant to ciprofloxacin. These findings are consistent with the scientific literature, which reports a high prevalence of such resistances in *Enterococcus* strains of animal origin, likely associated with the widespread use of tetracyclines and macrolides in livestock production (Arias and Murray, 2008). Resistance to chloramphenicol was also extremely high, probably resulting from co-selection phenomena. General features for the selected 42 isolates are reported in Supplementary Table S1.

Thirty-three out of thirty-four *E. faecalis* isolates were positive for the *optrA* gene alone (*n* = 30), or in association with *poxtA* and *cfr* (*n* = 2), or with *cfr* (*n* = 1); the only *optrA*-negative *E. faecalis* strain showed the *poxtA*–*cfr*(D) gene combination (Supplementary Table S1). It should be noted that the *optrA* gene was closely associated with the *E. faecalis* genetic background as it was found in almost all isolates of this species. These findings confirm the role of *E. faecalis* as a reservoir for the spread of *optrA* in the human setting (Deshpande et al., 2018; Brenciani et al., 2022). All *E. faecium* strains were positive for the *poxtA* gene and an isolate also contained the *optrA* gene (Supplementary Table S1). Despite the *poxtA* gene has previously been described in a MRSA of clinical origin from Italy (Antonelli et al., 2016, 2018), this linezolid resistance gene appears to be more common in *E. faecium* isolates of different origin (Brenciani et al., 2022). These results are consistent with previous reports of increasing *optrA* dissemination in *Enterococcus* from livestock and food products (Coccitto et al., 2023; Xuan et al., 2023; Fukuda et al., 2024). The high prevalence of optrA-positive *E. faecalis* observed in this study is in agreement with recent European reports describing optrA as the predominant transferable oxazolidinone resistance determinant in enterococci from livestock, meat products and related environments (Fioriti et al., 2020; Coccitto et al., 2022; Nüesch-Inderbinen et al., 2023a). Likewise, the detection of *poxtA* mainly in *E. faecium* isolates is consistent with previous findings from Italy and other European countries, where this gene has been increasingly reported in food-producing animals. Furthermore, most linezolid resistance determinants identified in the present study were associated with plasmids or other mobile genetic elements, confirming the major role of horizontal gene transfer in their dissemination. The contamination detected in slaughterhouse environmental samples and carcasses further supports the potential circulation of these resistant enterococci throughout the processing environment, in line with observations from other European food-production settings.

### Genomic analysis

Genome assemblies showed good overall quality, with genome sizes ranging from 2.5 to 3.2 Mb, N50 values between 63.7 and 597.2 kb, and average sequencing coverage ranging from 46.7 × to 111.8×, indicating overall good assembly quality suitable for downstream genomic analyses. To analyze the resistome, virulome, sequence type (ST), phylogenesis and the genetic context of oxazolidinone resistance determinants, all 42 selected enterococci were subjected to WGS analysis.

**Figure 2:**
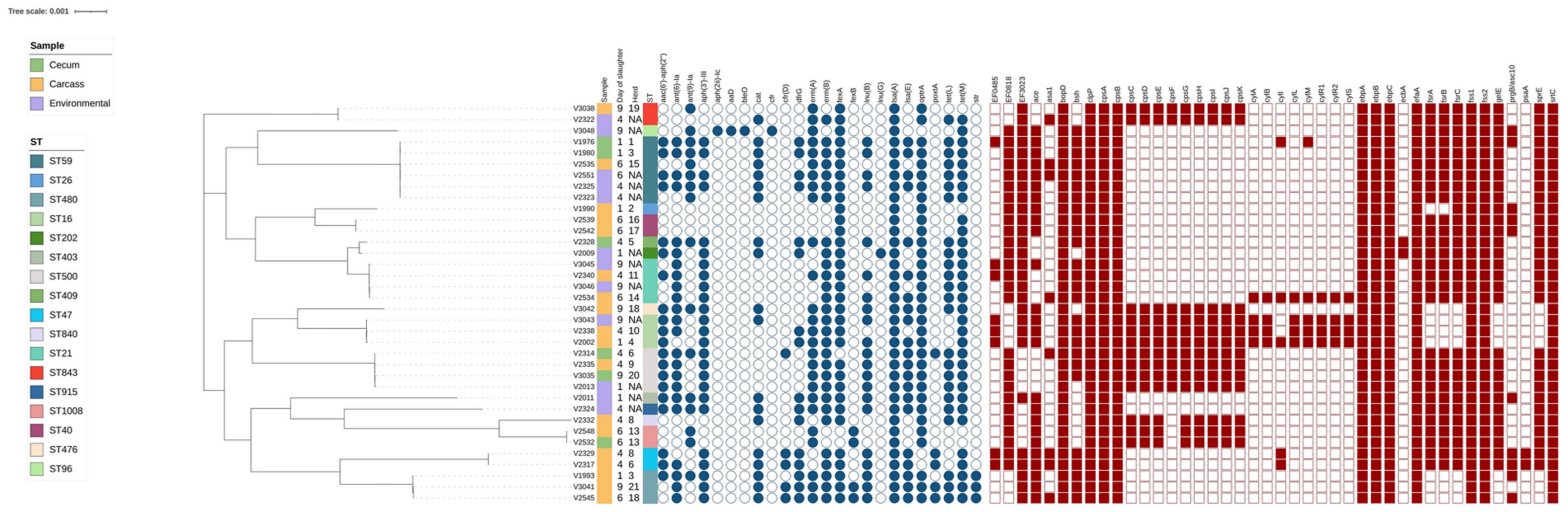
Phylogenetic tree annotated using iTOL (https://itol.embl.de/). The tree includes the 34 *E. faecalis* isolates analyzed in the present study. The farm and sample source (carcass, cecum, and environment), the slaughter day, the sequence type (ST), as well as the presence of antibiotic resistance genes and virulence factors for each isolate are represented in the phylogenetic tree.

**Figure 3:**
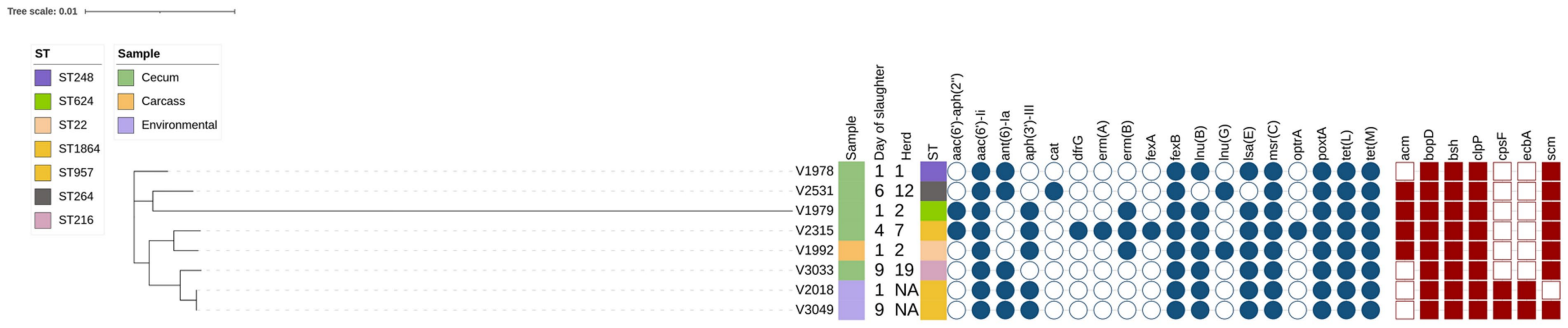
Phylogenetic tree annotated using iTOL (https://itol.embl.de/). The tree includes the 8 *E. faecium* isolates analyzed in the present study. The farm and sample source (carcass, cecum, and environment), the slaughter day, the sequence type (ST), as well as the presence of antibiotic resistance genes and virulence factors for each isolate are represented in the phylogenetic tree.

MLST revealed a high clonal diversity among the *E. faecalis* isolates recovered from the swine slaughterhouse, with 24 STs identified (Supplementary Table S1, Figures 2, 3). The predominance of ST59, ST21, and ST16 mirrored previous reports from animal and food-production settings (Fioriti et al., 2020; Freitas et al., 2020; Nüesch-Inderbinen et al., 2022), confirming their widespread occurrence at the human–animal–environment interface. Several clones detected in this study have been recognized as globally distributed lineages with clinical relevance, such as ST480, associated with hospital outbreaks and environmental sources (Ben Said et al., 2015; Mendes et al., 2018), and ST476, an *optrA*-carrying lineage reported in multiple reservoirs (Brenciani et al., 2024). High-risk clones like ST16, known for their zoonotic potential and multidrug resistance profiles (Novais et al., 2013; León-Sampedro et al., 2019), were also detected, underlining the potential public health implications of their circulation in swine production. In contrast, rare STs such as ST500, ST843, and ST96 were identified, for which no epidemiological information is currently available in PubMLST or PubMed, suggesting either a limited distribution or underreporting. Notably, isolates belonging to ST59, ST21, and ST40, lineages commonly linked to clinical infections as well as animal and food sources (Zischka et al., 2015; Elghaieb et al., 2020; Matle et al., 2023; Abdullahi et al., 2024) highlight the overlap between veterinary and human epidemiology. No clear clustering was observed by slaughter day or farm of origin, except for two clonal *E. faecalis* isolates from farm 13, both ST1008—one from the cecum and the other from the corresponding carcass. This result was also confirmed from the SNP analysis (Supplementary Tables S2, S3). This finding suggests potential cross-contamination during slaughter. *E. faecalis* ST1008, positive for *optrA*, has also been reported in raw pet food in several European countries (Freitas et al., 2021; Nüesch-Inderbinen et al., 2023a). The recovery of genetically indistinguishable isolates from the intestinal content and the corresponding carcass provides further evidence that slaughtering procedures may facilitate the transfer of resistant enterococci from animals to carcass surfaces. Although only a single clonal event was identified, this observation supports the role of slaughterhouses as critical control points where resistant bacteria can disseminate along the production chain. The detection of the same lineage in different animal-derived products across Europe further suggests the successful adaptation and circulation of this clone within food-associated environments.

Overall, ResFinder analysis revealed complex resistomes for the presence of several acquired antibiotic resistance genes beside linezolid resistance determinants (Table 3). Genes responsible for the resistance to aminoglycosides, macrolides, lincosamides, phenicols and tetracyclines were detected in almost all isolates (Table 3).

**Table 3:** Molecular typing, resistome, and virulome of the 42 enterococci investigated in this study.

Isolate	MIC LZD (mg/L)	Molecular typing	Acquired resistance genes									Acquired virulence genes						
			Aminoglycosides	Macrolides	Lincosamides	Tetracyclines	Streptogramins group B	Diaminopyrimidines	Amphenicols	Oxazolidinones	Bleomycin	Adhesins and surface proteins	Cytolysin	Biofilm formation	Pheromone response	Gelatinase	Hyaluronidase	Oxidative stress
*E. faecium* V1978	8	ST248	*aac(6′)*-Ii *ant(6)-*Ia	*msr*(C)	*lnu*(B) *lsa*(E)	*tet*(L) *tet*(M)	*lsa*(E)		*fexB* *poxtA*	*poxtA*		*efaAfm*						
*E. faecium* V1979	8	ST624	*aac(6′)*-Ii *aac(6′)-aph(2″)* *aph(3′)-*III	*erm*(B) *msr*(C)	*erm*(B) *lnu*(B) *lsa*(E)	*tet*(L) *tet*(M)	*erm*(B) *lsa*(E)		*fexB* *poxtA*	*poxtA*		*acm* *efaAfm*						
*E. faecium* V1992	4	ST22	*aac(6′)*-Ii *aph(3′)-*III	*erm*(B) *msr*(C)	*erm*(B) *lnu*(B) *lnu*(G) *lsa*(E)	*tet*(L) *tet*(M)	*erm*(B) *lsa*(E)		*fexB* *poxtA*	*poxtA*		*acm* *efaAfm*						
*E. faecium* V2018	4	ST1864	*aac(6′)*-Ii *ant(6)-*Ia *aph(3′)-*III	*msr*(C)	*lnu*(B) *lsa*(E)	*tet*(L) *tet*(M)	*lsa*(E)		*fexB* *poxtA*	*poxtA*		*efaAfm*						
*E. faecium* V2315	4	ST957	*aac(6′)*-Ii *aph(3′)-*III *aac(6′)-aph(2″)*	*msr*(C) *erm*(A) *erm*(B)	*lnu*(B) *lsa*(E) *erm*(A) *erm*(B)	*tet*(L) *tet*(M)	*lsa*(E) *erm*(B) *erm*(A)	*dfrG*	*fexA* *fexB* *optrA* *poxtA*	*optrA* *poxtA*		*acm* *efaAfm*						
*E. faecium* V2531	8	ST264	*aac(6′)*-Ii *ant(6)-*Ia	*msr*(C)	*lnu*(G)	*tet*(L) *tet*(M)			*fexB* *poxtA* *cat*	*poxtA*		*acm* *efaAfm*						
*E. faecium* V3033	4	ST216	*aac(6′)*-Ii *ant(6)-*Ia	*msr*(C)	*lnu*(B) *lsa*(E)	*tet*(L) *tet*(M)	*lsa*(E)		*fexB* *poxtA*	*poxtA*		*efaAfm*						
*E. faecium* V3049	4	ST1864	*aac(6′)*-Ii *ant(6)-*Ia *aph(3′)-*III	*msr*(C)	*lnu*(B) *lsa*(E)	*tet*(L) *tet*(M)	*lsa*(E)		*fexB* *poxtA*	*poxtA*		*efaAfm*						
*E. faecalis* V1976	8	ST59	*ant(9)-Ia,* *aac(6′)-aph(2″), ant(6)-*Ia, *aph(3′)-*III	*erm*(B) *erm*(A)	*lnu*(B) *lsa*(E) *lsa*(A) *erm*(B) *erm*(A)	*tet*(L) *tet*(M)	*lsa*(E) *erm*(B) *erm*(A)	*dfrG*	*optrA* *cat* *fexA*	*optrA*		*elrA, srtA, ace, efaAfs*		*fsrB*	*cCF10, cOB1* *cad, camE, ebpA, ebpB, ebpC*	*gelE*	*hylA* *hylB*	*tpx*
*E. faecalis* V1980	8	ST59	*ant(9)-Ia,* *aac(6′)-aph(2″), ant(6)-*Ia, *aph(3′)-*III	*erm*(B) *erm*(A)	*lnu*(B) *lsa*(E) *lsa*(A) *erm*(B) *erm*(A)	*tet*(L) *tet*(M)	*lsa*(E) *erm*(B) *erm*(A)	*dfrG*	*optrA* *cat* *fexA*	*optrA*		*elrA, srtA, ace, efaAfs*		*fsrB*	*cCF10, cOB1* *cad, camE, ebpA, ebpB, ebpC*	*gelE*	*hylA* *hylB*	*tpx*
*E. faecalis* V2323	8	ST59	*ant(9)-Ia*	*erm*(B) *erm*(A)	*lsa*(A) *erm*(B) *erm*(A)	*tet*(L) *tet*(M)	*erm*(B) *erm*(A)		*optrA* *cat* *fexA*	*optrA*		*elrA, srtA, ace, efaAfs*		*fsrB*	*cCF10, cOB1* *cad, camE, ebpA, ebpB, ebpC*	*gelE*	*hylA* *hylB*	*tpx*
*E. faecalis* V2325	8	ST59	*ant(9)-Ia,* *aac(6′)-aph(2″), ant(6)-*Ia, *aph(3′)-*III	*erm*(B) *erm*(A)	*lnu*(B) *lsa*(E) *lsa*(A) *erm*(B) *erm*(A)	*tet*(L) *tet*(M)	*lsa*(E) *erm*(B) *erm*(A)	*dfrG*	*optrA* *cat* *fexA*	*optrA*		*elrA, srtA, ace, efaAfs*		*fsrB*	*cCF10, cOB1* *cad, camE, ebpA, ebpB, ebpC*	*gelE*	*hylA* *hylB*	*tpx*
*E. faecalis* V2535	8	ST59	*ant(9)-Ia*	*erm*(B) *erm*(A)	*lsa*(A) *erm*(B) *erm*(A)	*tet*(L) *tet*(M)	*erm*(B) *erm*(A)		*optrA* *cat* *fexA*	*optrA*		*elrA, srtA, ace, efaAfs*		*fsrB*	*cCF10, cOB1* *cad, camE, ebpA, ebpB, ebpC*	*gelE*	*hylA* *hylB*	*tpx*
*E. faecalis* V2551	8	ST59	*ant(9)-Ia,* *aac(6′)-aph(2″), ant(6)-*Ia, *aph(3′)-*III	*erm*(B) *erm*(A)	*lnu*(B) *lsa*(E) *lsa*(A) *erm*(B) *erm*(A)	*tet*(L) *tet*(M)	*lsa*(E) *erm*(B) *erm*(A)	*dfrG*	*optrA* *cat* *fexA*	*optrA*		*elrA, srtA, ace, efaAfs*		*fsrB*	*cCF10, cOB1* *cad, camE, ebpA, ebpB, ebpC*	*gelE*	*hylA* *hylB*	*tpx*
*E. faecalis* V2340	8	ST21	*aac(6′)-aph(2″), ant(6)-*Ia, *aph(3′)-*III	*erm*(B) *erm*(A)	*lnu*(B) *lsa*(E) *lsa*(A) *erm*(B) *erm*(A)	*tet*(L) *tet*(M)	*lsa*(E) *erm*(B) *erm*(A)		*optrA* *fexA*	*optrA*		*elrA, srtA, ace, efaAfs*		*fsrB, agg*	*cCF10, cOB1* *cad, camE, ebpA, ebpB, ebpC*	*gelE*	*hylA* *hylB*	*tpx*
*E. faecalis* V2534	8	ST21	*ant(6)-*Ia, *aph(3′)-*III	*erm*(B)	*lnu*(B) *lsa*(E) *lsa*(A) *erm*(B)	*tet*(L) *tet*(M)	*lsa*(E) *erm*(B)		*optrA* *fexA*	*optrA*		*elrA, srtA, ace, efaAfs*	*cylA* *cylB* *cylL* *cylM*	*fsrB*	*cCF10, cOB1* *cad, camE* *ebpA, ebpB, ebpC*	*gelE*	*hylA* *hylB*	*tpx*
*E. faecalis* V3045	8	ST21	*ant(6)-*Ia, *aph(3′)-*III	*erm*(B)	*lsa*(A) *erm*(B)	*tet*(L) *tet*(M)	*erm*(B)		*optrA* *fexA*	*optrA*		*elrA, srtA, ace, efaAfs*		*fsrB, agg*	*cCF10, cOB1* *cad, camE* *ebpA, ebpB, ebpC*	*gelE*	*hylA* *hylB*	*tpx*
*E. faecalis* V3046	8	ST21	*ant(6)-*Ia, *aph(3′)-*III	*erm*(B)	*lsa*(A) *erm*(B)	*tet*(L) *tet*(M)	*erm*(B)		*optrA* *fexA*	*optrA*		*elrA, srtA, ace, efaAfs*		*fsrB*	*cCF10, cOB1* *cad, camE* *ebpA, ebpB, ebpC*	*gelE*	*hylA* *hylB*	*tpx*
*E. faecalis* V2013	8	ST500	*aac(6′)-aph(2″), ant(6)-*Ia, *aph(3′)-*III	*erm*(B) *erm*(A)	*lnu*(B) *lsa*(E) *lsa*(A) *erm*(B) *erm*(A)	*tet*(L) *tet*(M)	*lsa*(E) *erm*(B) *erm*(A)		*optrA* *fexA*	*optrA*		*elrA, srtA, ace, efaAfs*		*fsrB*	*cCF10, cOB1* *cad, camE* *ebpA, ebpB, ebpC*	*gelE*	*hylB*	*tpx*
*E. faecalis* V2335	8	ST500	*aac(6′)-aph(2″),* *ant(6)-*Ia, *aph(3′)-*III	*erm*(B) *erm*(A)	*lnu*(B) *lsa*(E) *lsa*(A) *erm*(B) *erm*(A)	*tet*(L) *tet*(M)	*lsa*(E) *erm*(B) *erm*(A)		*optrA* *fexA*	*optrA*		*elrA, srtA, ace, efaAfs*		*fsrB*	*cCF10, cOB1* *cad, camE* *ebpA, ebpB, ebpC*	*gelE*	*hylB*	*tpx*
*E. faecalis* V3035	8	ST500	*aac(6′)-aph(2″),* *ant(6)-*Ia, *aph(3′)-*III	*erm*(B) *erm*(A)	*lnu*(B) *lsa*(E) *lsa*(A) *erm*(B) *erm*(A)	*tet*(L) *tet*(M)	*lsa*(E) *erm*(B) *erm*(A)		*optrA* *fexA*	*optrA*		*elrA, srtA, ace, efaAfs*		*fsrB*	*cCF10, cOB1* *cad, camE* *ebpA, ebpB, ebpC*	*gelE*	*hylB*	*tpx*
*E. faecalis* V1993	>8	ST480	*ant(9)-Ia,* *aac(6′)-aph(2″), ant(6)-*Ia, *aph(3′)-*III *str*	*erm*(B) *erm*(A)	*lnu*(B) *lsa*(E) *lsa*(A) *erm*(B) *erm*(A)	*tet*(L) *tet*(M)	*lsa*(E) *erm*(B) *erm*(A)	*dfrG*	*optrA* *cat* *fexA*	*optrA*		*elrA, srtA, ace, efaAfs*		*agg*	*cCF10, cOB1* *cad, camE* *ebpA, ebpB, ebpC*		*hylA*	*tpx*
*E. faecalis* V2545	8	ST480	*ant(6)-*Ia, *aph(3′)-*III *str*	*erm*(B) *erm*(A)	*lnu*(B) *lsa*(E) *lsa*(A) *erm*(B) *erm*(A)	*tet*(L) *tet*(M)	*lsa*(E) *erm*(B) *erm*(A)	*dfrG*	*optrA* *poxtA* *cfr*(D) *cat* *fexA* *fexB*	*optrA* *poxtA* *cfr*(D)		*elrA, srtA, ace, efaAfs*		*agg*	*cCF10, cOB1* *cad, camE* *ebpA, ebpB, ebpC*		*hylA*	*tpx*
*E. faecalis* V3041	8	ST480	*ant(6)-*Ia, *aph(3′)-*III *str*	*erm*(B) *erm*(A)	*lnu*(B) *lsa*(E) *lsa*(A) *erm*(B) *erm*(A)	*tet*(L) *tet*(M)	*lsa*(E) *erm*(B) *erm*(A)	*dfrG*	*optrA* *poxtA* *cfr*(D) *cat* *fexA* *fexB*	*optrA* *poxtA* *cfr*(D)		*elrA, srtA, ace, efaAfs*		*agg*	*cCF10, cOB1* *cad, camE* *ebpA, ebpB, ebpC*		*hylA*	*tpx*
*E. faecalis* V2002	8	ST16	*aac(6′)-aph(2″),* *aph(3′)-*III	*erm*(B) *erm*(A)	*lnu*(B) *lsa*(E) *lsa*(A) *erm*(B) *erm*(A)	*tet*(M)	*lsa*(E) *erm*(B) *erm*(A)	*dfrG*	*optrA* *fexA*	*optrA*		*elrA, srtA, ace, efaAfs, espfm*	*cylA* *cylB* *cylL* *cylM*	*agg*	*cCF10, cOB1* *cad, camE* *ebpA, ebpB, ebpC*		*hylA*	*tpx*
*E. faecalis* V2338	8	ST16	*ant(6)-*Ia, *aph(3′)-*III, *aac(6′)-aph(2″)*	*erm*(B) *erm*(A)	*lsa*(A) *erm*(B) *erm*(A)	*tet*(M)	*erm*(B) *erm*(A)	*dfrG*	*optrA* *fexA*	*optrA*		*elrA, srtA, ace, efaAfs, espfs*	*cylA* *cylB* *cylL* *cylM*	*agg*	*cCF10, cOB1* *cad, camE* *ebpA, ebpB, ebpC*		*hylA*	*tpx*
*E. faecalis* V3043	8	ST16	*aac(6′)-aph(2″),* *aph(3′)-*III, *ant(6)-*Ia	*erm*(B) *erm*(A)	*lnu*(B) *lsa*(E) *lsa*(A) *erm*(B) *erm*(A)	*tet*(M)	*lsa*(E) *erm*(B) *erm*(A)		*optrA* *cat* *fexA*	*optrA*		*elrA, srtA, ace, efaAfs, espfs*	*cylA* *cylB* *cylL* *cylM*	*agg*	*cCF10, cOB1* *cad, camE* *ebpA, ebpB, ebpC*		*hylA*	*tpx*
*E. faecalis* V2539	8	ST40			*lsa*(A)	*tet*(M)			*optrA* *fexA*	*optrA*		*elrA, srtA, ace, efaAfs*		*fsrB, agg*	*cCF10, cOB1* *cad, camE* *ebpA, ebpB, ebpC*	*gelE*	*hylA* *hylB*	*tpx*
*E. faecalis* V2542	8	ST40			*lsa*(A)	*tet*(M)			*optrA* *fexA*	*optrA*		*elrA, srtA, ace, efaAfs*		*fsrB, agg*	*cCF10, cOB1* *cad, camE* *ebpA, ebpB, ebpC*	*gelE*	*hylA* *hylB*	*tpx*
*E. faecalis* V2322	8	ST843		*erm*(B) *erm*(A)	*lsa*(A) *erm*(B) *erm*(A)	*tet*(L) *tet*(M)	*erm*(B) *erm*(A)		*optrA* *cat* *fexA*	*optrA*		*elrA, srtA, ace, efaAfs*		*fsrB*	*cCF10, cOB1* *cad, camE* *ebpA, ebpB, ebpC*	*gelE*	*hylA*	*tpx*
*E. faecalis* V3038	8	ST843	*ant(9)-Ia*	*erm*(A)	*lsa*(A) *erm*(A)		*erm*(A)		*optrA* *fexA*	*optrA*		*elrA, srtA, ace, efaAfs*		*fsrB*	*cCF10, cOB1* *cad, camE* *ebpA, ebpB, ebpC*	*gelE*	*hylA*	*tpx*
*E. faecalis* V2532 *E. faecalis* V2548	4 4	ST1008	*ant(9)-Ia*	*erm*(A)	*lsa*(A) *erm*(A)		*erm*(A)		*optrA* *fexB*	*optrA*		*srtA, ace, efaAfs*		*fsrB*	*cCF10, cOB1* *cad, camE* *ebpA, ebpB, ebpC*	*gelE*	*hylB*	*tpx*
*E. faecalis* V2329	4	ST47	*aac(6′)-aph(2″),* *aph(3′)-*III	*erm*(B)	*lsa*(A) *erm*(B)	*tet*(M)	*erm*(B)	*dfrG*	*poxtA* *cfr*(D) *cat* *fexA*	*poxtA* *cfr*(D)		*elrA, srtA, ace, efaAfs*		*fsrB, agg*	*cCF10, cOB1* *cad, camE* *ebpA, ebpB, ebpC*	*gelE*	*hylA* *hylB*	*tpx*
*E. faecalis* V3042	8	ST476	*ant(9)-Ia,* *aac(6′)-aph(2″), ant(6)-*Ia, *aph(3′)-*III	*erm*(B) *erm*(A)	*lsa*(A) *erm*(B) *erm*(A)	*tet*(L) *tet*(M)	*erm*(B) *erm*(A)		*optrA* *cat* *fexA*	*optrA*		*elrA, srtA, ace, efaAfs*			*cCF10, cOB1* *cad, camE* *ebpA, ebpB, ebpC*		*hylA*	*tpx*
*E. faecalis* V1990	>8	ST26			*lsa*(A)				*optrA* *fexA*	*optrA*		*elrA, srtA, ace, efaAfs*		*agg*	*cCF10, cOB1* *cad, camE* *ebpA, ebpB, ebpC*	*gelE*	*hylA* *hylB*	*tpx*
*E. faecalis* V2009	4	ST202	*aac(6′)-aph(2″), ant(6)-*Ia, *aph(3′)-*III	*erm*(B)	*lsa*(A) *lnu*(G) *erm*(B)	*tet*(L) *tet*(M)	*erm*(B)	*dfrG*	*optrA* *cat* *fexA*	*optrA*		*elrA, srtA, ace, efaAfs*		*fsrB,*	*cCF10, cOB1* *cad, camE* *ebpA, ebpB, ebpC*	*gelE*	*hylA* *hylB*	*tpx*
*E. faecalis* V2011	8	ST403	*ant(9)-Ia,* *aac(6′)-aph(2″), ant(6)-*Ia, *aph(3′)-*III	*erm*(B) *erm*(A)	*lsa*(A) *lsa*(E) *lnu*(B) *erm*(B) *erm*(A)	*tet*(L) *tet*(M)	*lsa*(E) *erm*(B) *erm*(A)	*dfrG*	*optrA* *cat* *fexA*	*optrA*		*elrA, srtA, ace, efaAfs*		*fsrB, agg*	*cCF10, cOB1* *cad, camE* *ebpA, ebpB, ebpC*	*gelE*	*hylA* *hylB*	*tpx*
*E. faecalis* V2328	8	ST409	*ant(9)-Ia,* *aac(6′)-aph(2″), ant(6)-*Ia, *aph(3′)-*III	*erm*(B) *erm*(A)	*lsa*(A) *lsa*(E) *lnu*(B) *erm*(B) *erm*(A)	*tet*(L) *tet*(M)	*lsa*(E) *erm*(B) *erm*(A)	*dfrG*	*optrA* *cat* *fexA*	*optrA*		*elrA, srtA, ace, efaAfs*		*fsrB*	*cCF10, cOB1* *cad, camE* *ebpA, ebpB, ebpC*	*gelE*	*hylB*	*tpx*
*E. faecalis* V2332	4	ST840		*erm*(B)	*lsa*(A) *erm*(B)	*tet*(L) *tet*(M)	*erm*(B)	*dfrG*	*optrA* *cat* *fexA*	*optrA*		*srtA, ace, efaAfs*		*fsrB*	*cCF10, cOB1 cad, came, ebpA, ebpB, ebpC*	*gelE*	*hylB*	*tpx*
*E. faecalis* V2324	8	ST915	*ant(9)-Ia,* *aac(6′)-aph(2″), ant(6)-*Ia, *aph(3′)-*III	*erm*(B) *erm*(A)	*lnu*(B) *lsa*(E) *lsa*(A) *erm*(B) *erm*(A)	*tet*(L) *tet*(M)	*lsa*(E) *erm*(B) *erm*(A)	*dfrG*	*optrA* *cat* *fexA*	*optrA*		*srtA, ace, efaAfs*		*fsrB*	*cCF10, cOB1 cad, came, ebpA, ebpB, ebpC*	*gelE*	*hylB*	*tpx*
*E. faecalis* V3048	8	ST96	*ant(9)-Ia, aph(2″)-Ic, aadD*	*erm*(A)	*lsa*(A) *erm*(A)	*tet*(M)	*erm*(A)		*optrA* *cfr* *fexA*	*optrA* *cfr*	*bleO*	*elrA, srtA, ace, efaAfs*		*fsrB, agg*	*cCF10, cOB1* *cad, camE* *ebpA, ebpB, ebpC*	*gelE*	*hylA* *hylB*	*tpx*

Moreover, all strains exhibited several virulence genes encoding adhesins and surface proteins; *E. faecalis* isolates also showed several additional virulence factors involved in biofilm formation, gelatinase and hyaluronidases production, and sex pheromone and oxidative stress response (Table 3). No mutations involving the 23S rRNA or L3/L4 ribosomal proteins were detected either in the assembled genomes or using LRE-finder software (Supplementary Table S1).

### Genetic elements carrying oxazolidinone resistance genes

*E. faecium* and *E. faecalis* strains were arbitrarily clustered based on the genetic elements carrying linezolid resistance genes, whose main features are summarized in the Table 4. Interestingly, most of the genetic elements identified in both *E. faecium* and *E. faecalis* test strains had already been described in enterococci of different origins and from multiple countries. The p1818-c plasmid was first identified in *E. faecium* 1818 from human feces (Nüesch-Inderbinen et al., 2023a), while pAT47b-b was detected in *E. faecium* AT47b from pet food (Nüesch-Inderbinen et al., 2023a); both strains were isolated in Switzerland. Likewise, p17-318_1 had previously been reported in *E. faecium* 17–318 from a human digestive sample in France (Dejoies et al., 2021); notably, this *optrA*-carrying element contained several regions of virulence and resistance to antibiotics and heavy metals (Table 4). The pEgFS4-2 plasmid, on the other hand, was originally described in *E. gallinarum* FS4 from swine fecal samples in Italy (Coccitto et al., 2022), while pS46-3-a was previously identified in *E. hirae* S46-3 from swine feces in Switzerland (Nüesch-Inderbinen et al., 2023a), and p18-042_1 was first reported in *E. faecium* 18–042, isolated from a human digestive sample in France (Dejoies et al., 2021) (Table 4).

**Table 4:** Analysis of the genetic elements carrying oxazolidinone resistance genes of the 42 enterococci investigated in this study.

*E. faecium* and *E. faecalis* groups	Antibiotic resistance genes	Heavy metal resistance	Virulence	Genetic elements (GenBank accession no.)	Size (bp)	References
*E. faecium* V1978, and V1992	***poxtA*** ^**1**^ *, fexB*	-	-	p1818-c (CP091209)	23,864	Nüesch-Inderbinen et al. (2022)
*E. faecium* V2315, V3033, and V3049	***poxtA**, fexB, tet*(M)*, tet*(L)	-	-	pAT47b-b (CP097016)	41,159	Nüesch-Inderbinen et al. (2023a, 2023b)
*E. faecium* V2315	***optrA**, sat4, aac(6′)-aph(2″), ant(6)-Ia, ant(9)-Ia, aph(3′)-III, lsa*(E)*, lnu*(B)*, erm*(B)*, erm*(A) *and dfrG*	Iron, copper, mercury, cadmium	Fimbrial proteins	p17-318_1 (CP065771)	244,041	Dejoies et al. (2021)
*E. faecium* V1979	***poxtA**, fexB, tet*(M)*, tet*(L)	-	-	pEgFS4-2 (MZ291453)	38,387	Coccitto et al. (2022)
*E. faecium* V2018	***poxtA**, fexB, tet*(M)*, tet*(L)	-	-	pS46-3-a (CP088188)	35,172	Nüesch-Inderbinen et al. (2022)
*E. faecium* V2531	***poxtA***	-	-	p18-042_1 (CP066216)	9,392	Dejoies et al. (2021)
*E. faecalis* V1976, V1980, V2323, V2325, V2535, V2551, V3038, V2532, V2548, V3042, V2328 and V2324	***optrA**, fexA, erm*(A)*, ant(9)-Ia*	-	-	Tn*6674* (MK737778)	12,932	Li et al. (2019)
*E. faecalis* V1993, V2011 and V3048	***optrA**, fexA, erm*(A)*, ant(9)-Ia*	-	Surface exclusion protein Sea1/PrgA, aggregation substance Asa1/PrgB, pheromone response surface protein PrgC	pV1993-*optrA* (PX406248)	83,681	**This study**
*E. faecalis* V3048	***optrA**, **cfr**, ant(4′)-Ia, aph(2″)-IIIa, ble*	-	-	Chromosomal region (MZ291452)^2^	34,679	Coccitto et al. (2022)
*E. faecalis* V2335, V2340, V3035, V3045, V3043, V2539 and V2542	***optrA**, fexA, erm*(A)	-	Surface exclusion protein SEA1/PrgA, LPXTG-anchored aggregation substance	pAR_0780 (CP063981)	65,096	Unpublished
*E. faecalis* V2013, V2002, V2338	***optrA**, fexA*	-	-	pV1676-*optrA* (PP261343)	26,870	Cinthi et al. (2024)
*E. faecalis* V2332, V2545 and V3041	***optrA**, fexA, erm*(A)	-	Surface exclusion protein Sea1/PrgA, Aggregation substance Asa1/PrgB, Pheromone response surface protein PrgC, DNA-entry nuclease (Competence-specific nuclease)	pV2545-*optrA* (PX406249)	65,601	**This study**
*E. faecalis* V2545 and V3041	***poxtA**,* ***cfr*(D2)**, *fexB*	-	-	pEa_*cfr*(D2)-*poxtA* (OQ298927)	41,037	Coccitto et al. (2023)
*E. faecalis* V2534 and V3046	***optrA**, fexA*	-	-	pEFs17-1 (MT223178)	36,331	Yoon et al. (2020)
*E. faecalis* V1990	***optrA**, fexA*	-	Surface exclusion protein SEC10/PrgA, LPXTG-anchored aggregation subtance PrgB/Asc10	pAPT110 (MW012677)	53,177	Finisterra et al. (2021)
*E. faecalis* V2009	***optrA**, catA, tet*(M)*, tet*(L), *erm*(B)*, aph(3′)-IIIa, sat4, ant(6)-Ia, lnu*(B), *lsa*(E), *ant(9)-Ia, aph(2″)-Ia, dfrG*	Copper	-	p90_2023_A (CP127178)	92,090	Kelbert et al. (2023)
*E. faecalis* V2332	***optrA**, catA, fexA, tet*(M)*, tet*(L), *erm*(B), *aph(3′)-IIIa, sat4, ant(6)-Ia, aph(2″)-Ia*, *lsa*(E), *ant(9)-Ia, dfrG*	Copper	Bacterioncin	pV1461-*optrA*-like (PP003004)^3^	86,608	Coccitto et al. (2024)
*E. faecalis* V2329	***poxtA2, cfr*(D)** *, fexA*	-	-	pV386-like (MZ603802)^4^	16,310	Cinthi et al. (2022a, 2022b)

1 Oxazolidinone resistance genes are indicated in bold.

2 Chromosomal region (18 kb) identical to the corresponding segment of the *pEgFS4-1* plasmid (34,679 bp, accession no. MZ291452).

3 The *E. faecalis* V2332 isolate showed that *optrA* was on a 86,608-bp plasmid identical to the corresponding DNA region of the larger *pV1461-optrA* plasmid (112,928 bp, accession no. PP003004).

4 The *E. faecalis* V2329 isolate displayed that *poxtA2* and *cfr*(D) genes were co-located in a 16,310-bp plasmid identical to a DNA region of a larger *pV386* plasmid (33,480 bp, accession no. MZ603802).

Twelve *E. faecalis* isolates carried the *optrA* gene on the Tn*6674* transposon. This genetic element, typically inserted into the *radC* gene encoding a DNA repair protein, was first described in the porcine *E. faecalis* E1731 from China (Li et al., 2019) (Table 4).

However, in *E. faecalis* strains, the *optrA* gene was frequently associated with several previously described plasmids: pAR_0780, previously found in *E. faecalis* AR_0780, of unknown origin, from USA; pV1676-*optrA*, identified in *E. faecalis* V1676 from a wild boar fecal sample in Italy (Cinthi et al., 2024); pEFs17-1, detected in *E. faecalis* EFs17-1 of chicken origin in Republic of Korea (Yoon et al., 2020); pAPT110, first found in *E. faecalis* PF110 from raw-frozen dog food in Portugal (Freitas et al., 2021); p90_2023_A, recovered from *E. faecalis* 90_2023 in raw-meat sausage imported from Italy to Switzerland (Kelbert et al., 2023); and pV1461-*optrA*-like, described in *E. faecalis* V1461 from a swine sample collected in central Italy (Coccitto et al., 2024) (Table 4).

The *optrA* gene—associated with the *cfr* gene—was also found within a 18-kb chromosomal region identical to the corresponding segment of the pEgFS4-1 plasmid previously detected in *E. gallinarum* FS4 from swine faecal samples in Italy (Coccitto et al., 2022) (Table 4).

Two other previously described plasmids were found to carry the *poxtA*/*cfr*(D2) and *poxtA2*/*cfr*(D) genes in the *E. faecalis* tested strains: pEa_*cfr*(D2)-*poxtA*, originally identified in *E. avium* 44,917 from a swine brain (Coccitto et al., 2023) and pV386-like, previously detected in enterococci isolated from pig manure (Cinthi et al., 2022a) isolated from Italy (Table 4).

**Figure 4:**
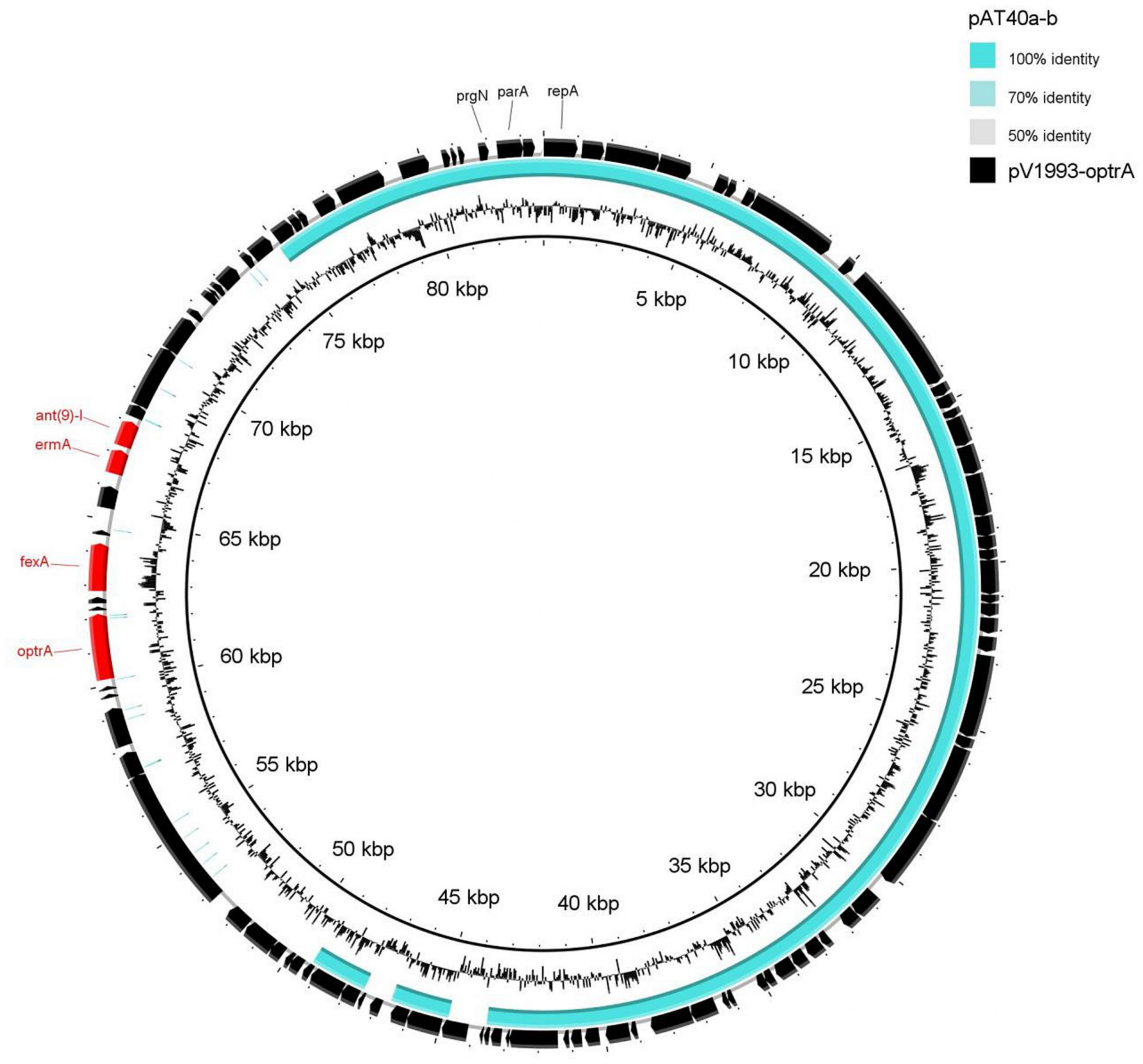
Circular maps of the pV1993-optrA plasmid (accession no. PX406248) in comparison with similar reported plasmids using BRIG software. Black arrows indicate the positions and orientations of genes; some antibiotic resistance determinants (red) and relevant genes described in this study are shown.

**Figure 5:**
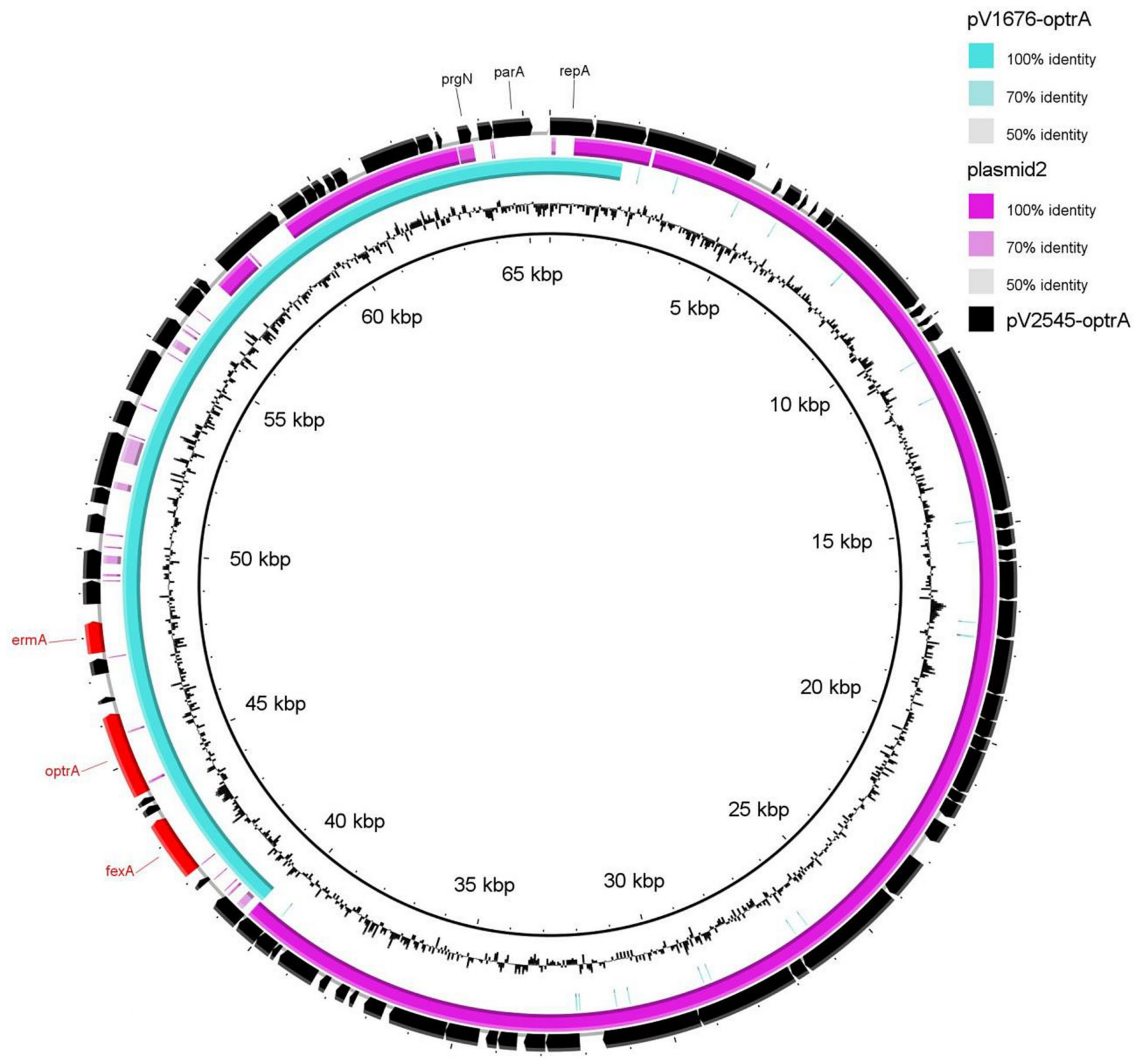
Circular maps of the pV2545-optrA plasmid (accession no. PX406249) in comparison with similar reported plasmids using BRIG software. Black arrows indicate the positions and orientations of genes; some antibiotic resistance determinants (red) and relevant genes described in this study are shown.

Although, as previously mentioned, the vast majority of genetic elements had already been described, this study identified in *E. faecalis* two novel *optrA*-carrying plasmids: pV1993-*optrA* and pV2545-*optrA* both belonging to the theta-replicating RepA_N replicon type. The pV1993-*optrA*, displayed 99.97% of nucleotide identity, but 67% of coverage, with the pAT40a-b plasmid (76,993 bp) detected in *E. faecalis* AT40a from pet food in Switzerland (Nüesch-Inderbinen et al., 2023b) (Figure 4). Interestingly, in one of the three isolates in which this plasmid was detected (V3048), another copy of the *optrA* associated with the *cfr* gene, was also identified on the chromosome (see above). The pV2545-*optrA* plasmid was instead the result of a fusion between two different plasmids: (*i*) the 67,319-bp plasmid 2 (99.37% DNA identity and 70% coverage) previously identified in *E. faecalis* 28157_4#78 of unknown origin from Netherlands; (*ii*) the 26,870-bp pV1676-*optrA* plasmid (99.98% DNA identity and 41% coverage), previously described in *E. faecalis* V1676 (Cinthi et al., 2024) (Figure 5) (Table 4). This newly characterised pV2545-*optrA* plasmid also harboured the *fexA* and *erm*(A) resistance genes.

A distinctive feature shared by all these genetic elements was the presence of several additional resistance genes—beyond those conferring resistance to oxazolidinones—which, through co-selection mechanisms, may have contributed to their dissemination and persistence.

## Conclusion

This study provides important insights into the carriage of oxazolidinone-resistant enterococci in the cecal contents of pigs at slaughter and their occurrence in the slaughterhouse environment and on carcasses. A major strength of this study is the integrated investigation of oxazolidinone-resistant enterococci across animal, carcass, and slaughterhouse environmental matrices, providing a comprehensive overview of their occurrence along the pig production chain. However, some limitations should be acknowledged, including the absence of human isolates, the lack of assessment of transmission to consumers, and the fact that bacterial persistence during food processing and cooking was not investigated.

A high prevalence of oxazolidinone-resistant enterococci was found in the gut of healthy pigs at slaughter. The presence of *optrA/poxtA* in enterococci in livestock may be favoured by the use of florfenicol on farms (Fukuda and Usui, 2025). In Italy, there was a drop in the overall annual sales of antibiotics in livestock from 371.0 mg/PCU in 2011 to 157.5 mg/PCU in 2022. However, sales of amphenicols increased from 1 mg/PCU to 2 mg/PCU in the same period (European Medicines Agency, 2023). The selective pressure generated by the use of amphenicols might explain the high prevalence of oxazolidinone-resistant enterococci found here.

Environmental sampling demonstrated that slaughterhouse premises became contaminated with oxazolidinone-resistant enterococci during processing, even though end-of-day cleaning and disinfection procedures were generally effective in reducing contamination. Notably, almost one third of carcasses was contaminated by oxazolidinone-resistant enterococci, suggesting a potential hazard for public health, indicating that these bacteria can persist along the slaughter process and reach carcass surfaces. Although pig caeca harboured a variety of *Enterococcus* species, *E. faecalis* predominated in carcasses and environmental samples, probably for its greater persistence compared with other members within the genus (Knysz et al., 2025).

The presence of high-risk clones carrying several antimicrobial resistance and virulence genes described in other geographical areas and from different hosts highlights the widespread distribution of oxazolidinone-resistant enterococci and their potential relevance within a One Health framework. Worryingly, the detection of the same genetic platforms carrying *optrA/poxtA* found in clones of various origins suggests the circulation of related resistance determinants across different ecological niches, although transmission pathways were not investigated in the present study. Overall, these findings suggest that the pig production chain may contribute to the maintenance and circulation of oxazolidinone-resistant enterococci, warranting further investigations into their transmission dynamics and potential public health significance (Almeida-Santos et al., 2025).

Slaughterhouses play a pivotal role in the food chain and represent a hotspot for the transmission and dissemination of antibiotic resistances. An integrated approach—encompassing prudent antibiotic use in livestock, good hygiene practices, and increased awareness of the problem will be essential to mitigate the spread of antimicrobial resistance along the food production chain.

Future studies should adopt a One Health approach integrating animal, food, environmental, and human data to better understand transmission dynamics and quantify the public health relevance of these resistant bacteria.

## Data Availability

The data set presented in this study can be found in the NCBI Sequence Read Archive (SRA) under BioProject PRJNA1295837 (SAMN50187228–SAMN50187269).

## Footnotes

1
https://github.com/rrwick/Unicycler

2 https://pubmlst.org/organisms/enterococcus-faecalis, https://pubmlst.org/organisms/enterococcus-faecium

3
https://blast.ncbi.nlm.nih.gov/BlastAlign.cgi

4
https://rast.nmpdr.org/

## References

[ref1] Abdullahi I. N. Lozano C. Zarazaga M. Latorre-Fernández J. Hallstrøm S. Rasmussen A. . (2024). Genomic characterization and phylogenetic analysis of linezolid-resistant Enterococcus from the nostrils of healthy hosts identifies zoonotic transmission. Curr. Microbiol. 81:225. doi: 10.1007/s00284-024-03737-2, 38877167 PMC11178607

[ref2] Almeida-Santos A. C. Duarte B. Tedim A. P. Teixeira M. J. Prata J. C. Azevedo R. M. S. . (2025). The healthy human gut can take it all: vancomycin-variable, linezolid-resistant strains and specific bacteriocin-species interplay in Enterococcus spp. Appl. Environ. Microbiol. 91:e0169924. doi: 10.1128/aem.01699-24, 39699199 PMC11784074

[ref3] Antonelli A. D’Andrea M. M. Brenciani A. Galeotti C. L. Morroni G. Pollini S. . (2018). Characterization of poxtA, a novel phenicol-oxazolidinone-tetracycline resistance gene from an MRSA of clinical origin. J. Antimicrob. Chemother. 73, 1763–1769. doi: 10.1093/jac/dky088, 29635422

[ref4] Antonelli A. D’Andrea M. M. Galano A. Borchi B. Brenciani A. Vaggelli G. . (2016). Linezolid-resistant *cfr*-positive MRSA, Italy. J. Antimicrob. Chemother. 71, 2349–2351. doi: 10.1093/jac/dkw108 27073268

[ref5] Arias C. A. Murray B. E. (2008). Emergence and management of drug-resistant enterococcal infections. Expert Rev. Anti-Infect. Ther. 6, 637–655. doi: 10.1586/14787210.6.5.637, 18847403

[ref6] Arias C. A. Murray B. E. (2012). The rise of the Enterococcus: beyond vancomycin resistance. Nat. Rev. Microbiol. 10, 266–278. doi: 10.1038/nrmicro2761, 22421879 PMC3621121

[ref7] Ben Said L. Klibi N. Lozano C. Dziri R. Ben Slama K. Boudabous A. . (2015). Diversity of enterococcal species and characterization of high-level aminoglycoside resistant enterococci of samples of wastewater and surface water in Tunisia. Sci. Total Environ. 531, 11–17. doi: 10.1016/j.scitotenv.2015.05.091, 26026404

[ref8] Brenciani A. Cinthi M. Coccitto S. N. Massacci F. R. Albini E. Cucco L. . (2024). Global spread of the linezolid-resistant *Enterococcus faecalis* ST476 clonal lineage carrying *optrA*. J. Antimicrob. Chemother. 79, 846–850. doi: 10.1093/jac/dkae039, 38366373

[ref9] Brenciani A. Morroni G. Schwarz S. Giovanetti E. (2022). Oxazolidinones: mechanisms of resistance and mobile genetic elements involved. J. Antimicrob. Chemother. 77, 2596–2621. doi: 10.1093/jac/dkac263, 35989417

[ref10] Cinthi M. Coccitto S. N. D’Achille G. Morroni G. Simoni S. Fioriti S. . (2022a). Characterization of a novel *cfr*(D)/*poxtA*-carrying plasmid in an oxazolidinone-resistant *Enterococcus casseliflavus* isolate from swine manure, Italy. J. Glob. Antimicrob. Resist. 30, 308–310. doi: 10.1016/j.jgar.2022.07.007 35830951

[ref11] Cinthi M. Coccitto S. N. Fioriti S. Morroni G. Simoni S. Vignaroli C. . (2022b). Occurrence of a plasmid co-carrying *cfr*(D) and *poxtA2* linezolid resistance genes in *Enterococcus faecalis* and *Enterococcus casseliflavus* from porcine manure, Italy. J. Antimicrob. Chemother. 77, 598–603. doi: 10.1093/jac/dkab456, 34910146

[ref12] Cinthi M. Coccitto S. N. Massacci F. R. Albini E. Binucci G. Gobbi M. . (2024). Genomic analysis of enterococci carrying optrA, poxtA, and vanA resistance genes from wild boars, Italy. J. Appl. Microbiol. 135:lxae193. doi: 10.1093/jambio/lxae193, 39076010

[ref13] CLSI (2024a). CLSI M100- Performance Standards for Antimicrobial Susceptibility Testing - 34th Edition. CLSI.

[ref14] CLSI (2024b). CLSI VET01S - Performance Standards for Antimicrobial Disk and Diluition Susceptibility Tests for Bacteria Isolated From Animals - 7th Edition. CLSI.

[ref15] Coccitto S. N. Cinthi M. Fioriti S. Morroni G. Simoni S. Vignaroli C. . (2022). Linezolid-resistant *Enterococcus gallinarum* isolate of swine origin carrying cfr, optrA and poxtA genes. J. Antimicrob. Chemother. 77, 331–337. doi: 10.1093/jac/dkab408, 35076077

[ref16] Coccitto S. N. Cinthi M. Massacci F. R. Albini E. Magistrali C. F. Brenciani A. . (2024). Genetic elements harbouring oxazolidinone resistance genes detected in swine enterococci circulate in clinical isolates, Italy. J. Glob. Antimicrob. Resist. 38, 245–246. doi: 10.1016/j.jgar.2024.06.016 39004341

[ref17] Coccitto S. N. Cinthi M. Simoni S. Vignaroli C. Massacci F. R. Albini E. . (2023). Identification of plasmids co-carrying cfr(D)/optrA and cfr(D2)/poxtA linezolid resistance genes in two *Enterococcus avium* isolates from swine brain. Vet. Microbiol. 282:109749. doi: 10.1016/j.vetmic.2023.109749, 37116421

[ref18] Dejoies L. Sassi M. Schutz S. Moreaux J. Zouari A. Potrel S. . (2021). Genetic features of the poxtA linezolid resistance gene in human enterococci from France. J. Antimicrob. Chemother. 76, 1978–1985. doi: 10.1093/jac/dkab116 33895846

[ref19] Deshpande L. M. Castanheira M. Flamm R. K. Mendes R. E. (2018). Evolving oxazolidinone resistance mechanisms in a worldwide collection of enterococcal clinical isolates: results from the SENTRY antimicrobial surveillance program. J. Antimicrob. Chemother. 73, 2314–2322. doi: 10.1093/jac/dky188, 29878213

[ref20] EFSA (2024). The European Union summary report on antimicrobial resistance in zoonotic and indicator bacteria from humans, animals and food in 2021–2022. EFSA J. 22, 1–195. doi: 10.2903/j.efsa.2024.8583 PMC10900121 38419967

[ref21] Elghaieb H. Tedim A. P. Abbassi M. S. Novais C. Duarte B. Hassen A. . (2020). From farm to fork: identical clones and Tn6674-like elements in linezolid-resistant *Enterococcus faecalis* from food-producing animals and retail meat. J. Antimicrob. Chemother. 75, 30–35. doi: 10.1093/jac/dkz419, 31605129

[ref22] European Medicines Agency (2023) European sales and use of Antimicrobials for Veterinary medicine - Annual Surveillance Report for 2023.

[ref23] Fioriti S. Coccitto S. N. Cedraro N. Simoni S. Morroni G. Brenciani A. . (2021). Linezolid resistance genes in enterococci isolated from sediment and zooplankton in two Italian coastal areas. Appl. Environ. Microbiol. 87, e02958–20. doi: 10.1128/AEM.02958-20, 33608287 PMC8091008

[ref9001] Finisterra L. Duarte B. Peixe L. Novais C. Freitas A. R. (2021). Industrial dog food is a vehicle of multidrug-resistant enterococci carrying virulence genes often linked to human infections. Int J Food Microbiol. 358:109284. doi: 10.1016/j.ijfoodmicro.2021.109284, 34144837

[ref24] Fioriti S. Morroni G. Coccitto S. N. Brenciani A. Antonelli A. Di Pilato V. . (2020). Detection of oxazolidinone resistance genes and characterization of genetic environments in enterococci of swine origin, Italy. Microorganisms 8:e02758–20. doi: 10.3390/microorganisms8122021, 33348682 PMC7766396

[ref25] Freitas A. R. Finisterra L. Tedim A. P. Duarte B. Novais C. Peixe L. (2021). Linezolid- and multidrug-resistant enterococci in raw commercial dog food, Europe, 2019-2020. Emerg. Infect. Dis. 27, 2221–2224. doi: 10.3201/eid2708.204933, 34287135 PMC8314808

[ref26] Freitas A. R. Tedim A. P. Novais C. Lanza V. F. Peixe L. (2020). Comparative genomics of global *optrA*-carrying *Enterococcus faecalis* uncovers a common chromosomal hotspot for *optrA* acquisition within a diversity of core and accessory genomes. Emerg Infect Dis. 27:2221–2224. doi: 10.1099/mgen.0.000350 PMC7371108 32149599

[ref27] Fukuda A. Nakajima C. Suzuki Y. Usui M. (2024). Transferable linezolid resistance genes (optrA and poxtA) in enterococci derived from livestock compost at Japanese farms. J. Glob. Antimicrob. Resist. 36, 336–344. doi: 10.1016/j.jgar.2024.01.022, 38336229

[ref28] Fukuda A. Usui M. (2025). Selection and maintenance of mobile linezolid-resistance genes and plasmids carrying them in the presence of florfenicol, an animal-specific antimicrobial. Access Microbiol. 36:336–344. doi: 10.1099/acmi.0.000997.v3, 40698117 PMC12282029

[ref29] Kelbert L. Stevens M. J. A. Horlbog J. A. Biggel M. Stephan R. (2023). Completely assembled genome sequence of the florfenicol-resistant *Enterococcus faecalis* strain 90_2023 isolated from a raw sausage imported from Italy to Switzerland. Microbiol. Resour. Announc. 12:e0061023. doi: 10.1128/MRA.00610-23, 37729579 PMC10586136

[ref30] Knysz P. Szkucik K. Gondek M. (2025). Rabbit carcasses as important vectors of multidrug-resistant *Enterococcus faecalis*, but not *E. faecium*: prevalence and molecular characterization from a study in Poland. BMC Vet. Res. 21:400. doi: 10.1186/s12917-025-04849-y, 40468370 PMC12135610

[ref31] Koutsoumanis K. Allende A. Álvarez-Ordóñez A. Bolton D. Bover-Cid S. Chemaly M. . (2022). Transmission of antimicrobial resistance (AMR) during animal transport. EFSA J. Eur. Food Saf. Auth. 20:e07586. doi: 10.2903/j.efsa.2022.7586 PMC9593722 36304831

[ref32] León-Sampedro R. Del Campo R. Rodriguez-Baños M. Lanza V. F. Pozuelo M. J. Francés-Cuesta C. . (2019). Phylogenomics of *Enterococcus faecalis* from wild birds: new insights into host-associated differences in core and accessory genomes of the species. Environ. Microbiol. 21, 3046–3062. doi: 10.1111/1462-2920.14702, 31162871

[ref33] Li D. Li X.-Y. Schwarz S. Yang M. Zhang S.-M. Hao W. . (2019). Tn6674 is a novel enterococcal optrA-carrying multiresistance transposon of the Tn554 family. Antimicrob. Agents Chemother. 63:e00809–19. doi: 10.1128/AAC.00809-19, 31209008 PMC6709469

[ref34] Matle I. Atanda A. C. Pierneef R. Magwedere K. Mafuna T. (2023). Resistome, mobilome, virulome analysis and phylogenomics of *Enterococcus faecalis* isolated from raw muscle foods of beef origin in Gauteng, South Africa. Genomics 115:110742. doi: 10.1016/j.ygeno.2023.110742, 37967685

[ref35] Mendes R. E. Deshpande L. Streit J. M. Sader H. S. Castanheira M. Hogan P. A. . (2018). ZAAPS programme results for 2016: an activity and spectrum analysis of linezolid using clinical isolates from medical centres in 42 countries. J. Antimicrob. Chemother. 73, 1880–1887. doi: 10.1093/jac/dky099, 29659858

[ref36] Morroni G. Brenciani A. Antonelli A. D’Andrea M. M. Di Pilato V. Fioriti S. . (2018). Characterization of a multiresistance plasmid carrying the optrA and cfr resistance genes from an *Enterococcus faecium* clinical isolate. Front. Microbiol. 9:2189. doi: 10.3389/fmicb.2018.02189, 30271398 PMC6142821

[ref37] Novais C. Freitas A. R. Silveira E. Antunes P. Silva R. Coque T. M. . (2013). Spread of multidrug-resistant Enterococcus to animals and humans: an underestimated role for the pig farm environment. J. Antimicrob. Chemother. 68, 2746–2754. doi: 10.1093/jac/dkt289, 23861310

[ref38] Nüesch-Inderbinen M. Biggel M. Haussmann A. Treier A. Heyvaert L. Cernela N. . (2023a). Oxazolidinone resistance genes in florfenicol-resistant enterococci from beef cattle and veal calves at slaughter. Front. Microbiol. 14:1150070. doi: 10.3389/fmicb.2023.1150070, 37389336 PMC10301837

[ref39] Nüesch-Inderbinen M. Haussmann A. Treier A. Zurfluh K. Biggel M. Stephan R. (2022). Fattening pigs are a reservoir of florfenicol-resistant enterococci Harboring oxazolidinone resistance genes. J. Food Prot. 85, 740–746. doi: 10.4315/JFP-21-431, 35258564

[ref40] Nüesch-Inderbinen M. Heyvaert L. Treier A. Zurfluh K. Cernela N. Biggel M. . (2023b). High occurrence of *Enterococcus faecalis*, *Enterococcus faecium*, and *Vagococcus lutrae* harbouring oxazolidinone resistance genes in raw meat-based diets for companion animals - a public health issue, Switzerland, September 2018 to may 2020. Euro Surveill. Bull. Eur. sur les Mal. Transm. 28:2200496. doi: 10.2807/1560-7917.ES.2023.28.6.2200496 PMC9912375 36757316

[ref41] Page A. J. Cummins C. A. Hunt M. Wong V. K. Reuter S. Holden M. T. G. . (2015). Roary: rapid large-scale prokaryote pan genome analysis. Bioinformatics 31, 3691–3693. doi: 10.1093/bioinformatics/btv421, 26198102 PMC4817141

[ref42] Price M. N. Dehal P. S. Arkin A. P. (2010). FastTree 2 - approximately maximum-likelihood trees for large alignments. PLoS One 5:e9490. doi: 10.1371/journal.pone.0009490, 20224823 PMC2835736

[ref43] Prjibelski A. Antipov D. Meleshko D. Lapidus A. Korobeynikov A. (2020). Using SPAdes de novo assembler. Curr. Protoc. Bioinform. 70:e102. doi: 10.1002/cpbi.102, 32559359

[ref44] Sadowy E. Luczkiewicz A. (2014). Drug-resistant and hospital-associated *Enterococcus faecium* from wastewater, riverine estuary and anthropogenically impacted marine catchment basin. BMC Microbiol. 14:66. doi: 10.1186/1471-2180-14-66, 24629030 PMC4004213

[ref45] Savin M. Bierbaum G. Andre J. Heinemann C. Parcina M. Sib E. . (2020a). Antibiotic-resistant bacteria and antimicrobial residues in wastewater and process water from German pig slaughterhouses and their receiving municipal wastewater treatment plants. Sci. Total Environ. 727:138788. doi: 10.1016/j.scitotenv.2020.138788 32498197

[ref46] Savin M. Bierbaum G. Hammerl A. Heinemann C. Parcina M. Sib E. (2020b). ESKAPE Bacteria and extended-Spectrum-beta-lactamase- producing *Escherichia coli* isolated from wastewater and process water from German poultry slaughterhouses. Appl. Env. Microbiol. 86, 1–18. doi: 10.1128/AEM.02748-19, 32033950 PMC7117925

[ref47] Schwarz S. Zhang W. Du X.-D. Krüger H. Feßler A. T. Ma S. . (2021). Mobile oxazolidinone resistance genes in gram-positive and gram-negative bacteria. Clin. Microbiol. Rev. 34:e0018820. doi: 10.1128/CMR.00188-20, 34076490 PMC8262807

[ref48] Seemann T. (2014). Prokka: rapid prokaryotic genome annotation. Bioinformatics 30, 2068–2069. doi: 10.1093/bioinformatics/btu153, 24642063

[ref49] Wang Y. Li X. Fu Y. Chen Y. Wang Y. Ye D. . (2020). Association of florfenicol residues with the abundance of oxazolidinone resistance genes in livestock manures. J. Hazard. Mater. 399:123059. doi: 10.1016/j.jhazmat.2020.123059, 32516648

[ref50] Wee B. A. Muloi D. M. van Bunnik B. A. D. (2020). Quantifying the transmission of antimicrobial resistance at the human and livestock interface with genomics. Clin. Microbiol. Infect. Off. Publ. Eur. Soc. Clin. Microbiol. Infect. Dis. 26, 1612–1616. doi: 10.1016/j.cmi.2020.09.019, 32979568 PMC7721588

[ref51] Xuan H. Xia L. Schwarz S. Jia H. Yao X. Wang S. . (2023). Various mobile genetic elements carrying optrA in Enterococcus faecium and *Enterococcus faecalis* isolates from swine within the same farm. J. Antimicrob. Chemother. 78, 504–511. doi: 10.1093/jac/dkac421, 36508313

[ref52] Yoon S. Son S. H. Kim Y. B. Seo K. W. Lee Y. J. (2020). Molecular characteristics of optrA-carrying *Enterococcus faecalis* from chicken meat in South Korea. Poult. Sci. 99, 6990–6996. doi: 10.1016/j.psj.2020.08.062, 33248615 PMC7704738

[ref53] Zischka M. Künne C. T. Blom J. Wobser D. Sakιnç T. Schmidt-Hohagen K. . (2015). Comprehensive molecular, genomic and phenotypic analysis of a major clone of *Enterococcus faecalis* MLST ST40. BMC Genomics 16:175. doi: 10.1186/s12864-015-1367-x, 25887115 PMC4374294

